# Point-prevalence survey of antibiotic use at three public referral hospitals in Kenya

**DOI:** 10.1371/journal.pone.0270048

**Published:** 2022-06-16

**Authors:** Sylvia Omulo, Margaret Oluka, Loice Achieng, Eric Osoro, Rosaline Kinuthia, Anastasia Guantai, Sylvia Adisa Opanga, Marion Ongayo, Linus Ndegwa, Jennifer R. Verani, Eveline Wesangula, Jarred Nyakiba, Jones Makori, Wilson Sugut, Charles Kwobah, Hanako Osuka, M. Kariuki Njenga, Douglas R. Call, Guy H. Palmer, Daniel VanderEnde, Ulzii-Orshikh Luvsansharav

**Affiliations:** 1 Paul G. Allen School for Global Health, Washington State University, Pullman, WA, United States of America; 2 Washington State University Global Health-Kenya, Nairobi, Kenya; 3 University of Nairobi Institute of Tropical and Infectious Diseases, Nairobi, Kenya; 4 Department of Pharmacology and Pharmacognosy, School of Pharmacy, University of Nairobi, Nairobi, Kenya; 5 Department of Clinical Medicine and Therapeutics, University of Nairobi, Nairobi, Kenya; 6 Kenyatta National Hospital, Nairobi, Kenya; 7 Department of Pharmaceutics and Pharmacy Practice, School of Pharmacy, University of Nairobi, Nairobi, Kenya; 8 Mbagathi County Hospital, Nairobi, Kenya; 9 Division of Global Health Protection, US Centers for Disease Control and Prevention, Nairobi, Kenya; 10 Patient and Health Workers Safety Unit, Ministry of Health, Nairobi, Kenya; 11 Coast Provincial General Hospital, Mombasa, Kenya; 12 Moi Teaching and Referral Hospital, Eldoret, Kenya; 13 Department of Medicine, Moi University School of Medicine, Eldoret, Kenya; 14 National Center for Emerging and Zoonotic Infectious Diseases, US Centers for Disease Control and Prevention, Atlanta, GA, United States of America; LSU Health Shreveport, UNITED STATES

## Abstract

Antimicrobial stewardship encourages appropriate antibiotic use, the specific activities of which will vary by institutional context. We investigated regional variation in antibiotic use by surveying three regional public hospitals in Kenya. Hospital-level data for antimicrobial stewardship activities, infection prevention and control, and laboratory diagnostic capacities were collected from hospital administrators, heads of infection prevention and control units, and laboratory directors, respectively. Patient-level antibiotic use data were abstracted from medical records using a modified World Health Organization point-prevalence survey form. Altogether, 1,071 consenting patients were surveyed at Kenyatta National Hospital (KNH, n = 579), Coast Provincial General Hospital (CPGH, n = 229) and Moi Teaching and Referral Hospital (MTRH, n = 263). The majority (67%, 722/1071) were ≥18 years and 53% (563/1071) were female. Forty-six percent (46%, 489/1071) were receiving at least one antibiotic. Antibiotic use was higher among children <5 years (70%, 150/224) than among other age groups (40%, 339/847; *P* < 0.001). Critical care (82%, 14/17 patients) and pediatric wards (59%, 155/265) had the highest proportion of antibiotic users. Amoxicillin/clavulanate was the most frequently used antibiotic at KNH (17%, 64/383 antibiotic doses), and ceftriaxone was most used at CPGH (29%, 55/189) and MTRH (31%, 57/184). Forty-three percent (326/756) of all antibiotic prescriptions had at least one missed dose recorded. Forty-six percent (204/489) of patients on antibiotics had a specific infectious disease diagnosis, of which 18% (37/204) had soft-tissue infections, 17% (35/204) had clinical sepsis, 15% (31/204) had pneumonia, 13% (27/204) had central nervous system infections and 10% (20/204) had obstetric or gynecological infections. Of these, 27% (56/204) had bacterial culture tests ordered, with culture results available for 68% (38/56) of tests. Missed antibiotic doses, low use of specimen cultures to guide therapy, high rates of antibiotic use, particularly in the pediatric and surgical population, and preference for broad-spectrum antibiotics suggest antibiotic use in these tertiary care hospitals is not optimal. Antimicrobial stewardship programs, policies, and guidelines should be tailored to address these areas.

## Introduction

Antimicrobial resistance (AMR) is a major public health concern that is global in scope [1, 2]. It is estimated that infections due to antimicrobial-resistant organisms are associated with approximately 50,000 deaths annually in Europe and the US alone [3] and that globally there are an estimated 214,000 neonatal deaths annually due to antimicrobial-resistant pathogens [4]. Less is known about the impact of this problem in African countries [5] although the burden is thought to be high.

Overuse and misuse of antimicrobials are considered important drivers of AMR. In the United States, about 30% of antimicrobial therapy is considered inappropriate [6]. In African countries, these estimates exceed 45%, and vary within primary and tertiary care facilities [7, 8]. Thus, healthcare facilities where antimicrobials are frequently used, are high-risk settings for the selection and spread of resistant bacteria [9]. Antimicrobial stewardship (AMS) programs aimed at optimizing antibiotic use have been implemented successfully in high-income countries without increasing healthcare costs [10, 11]. Nevertheless, there can be significant variation in antibiotic use among facilities [12], with wider variations observed between facilities that experience different rates of healthcare-associated infections, and that serve populations with different disease burdens. In low- and middle-income countries where the burden of antimicrobial resistance is presumably high [13] and access to antibiotics largely unregulated [5]. Data of this nature are particularly important for countries that are in the process of developing and implementing national antimicrobial-resistance action plans [14].

Antibiotic prescribing patterns and antimicrobial stewardship efforts in Kenya are not widely documented. We conducted a point-prevalence survey to describe the antibiotic use prevalence, common antimicrobial drug types, prescribing patterns, and indications for antibiotic use among inpatients, and existing AMS activities in three large public referral hospitals located in three geographically distinct regions of Kenya.

## Materials and methods

This point-prevalence survey was conducted at Kenyatta National Hospital (KNH, in September 2017, and at Coast Provincial General Hospital (CPGH) and at Moi Teaching and Referral Hospital (MTRH) in March–April 2018. According to the WHO protocol for point-prevalence studies [15]—which was applied for this survey—KNH and MTRH are tertiary hospitals while CPGH is a secondary hospital. Patients were eligible if admitted before 8 a.m.; patients from the following wards were not eligible: labor and delivery, radiology, rehabilitation, and short-stay wards. Neonates born and admitted before 8 a.m. were included and considered as individuals separate from their mothers. For each day of the survey, a census enumerated the total number of patients within a selected ward. The first enrollee was randomly selected from the first two (CPGH) or three (KNH and MTRH) listed patients from the census, proportional to estimated inpatient bed capacities. Subsequent survey participants were identified by systematically selecting every second or third patient, respectively, with the former used for hospitals having 250–700 total inpatient beds and the latter used for hospitals having >700 beds [15]. Informed consent was obtained from participants or their guardians before enrollment and before accessing their medical records for data abstraction. Patients (or their guardians) who declined participation were replaced by the next eligible patient on the sampling list.

Modified versions of the World Health Organization (WHO) point-prevalence survey (PPS) forms [15] were used to collect hospital (S1 File) and patient-level data (S2 File). Hospital-level data were collected by the survey coordinator who conducted in-person interviews with hospital administrators, heads of infection prevention and control units, and laboratory department heads regarding ongoing antimicrobial stewardship activities, existing infection prevention and control programs, and microbiology diagnostic capacities. Patient-level data were collected by a two-member team composed of a clinical or medical officer, and a pharmacist. The teams abstracted data from patient medical records, treatment sheets and nurses’ notes. These data included patient demographics, healthcare exposures, antibiotics used, diagnoses for which the current antibiotics were administered, and culture and susceptibility tests conducted during the current admission.

When not explicitly indicated, antibiotic use for prophylaxis was inferred for patients who had undergone surgeries for which prophylactic antibiotics are recommended, and for HIV-infected patients receiving co-trimoxazole. Diagnoses/indications for which antibiotics were prescribed were recorded following prespecified PPS indication codes, S2 File. Antibiotic use for prophylaxis or for unspecified infectious conditions was excluded from detailed analyses as these were interpreted differently between the two survey periods (2017 and 2018). Antibiotics prescribed primarily for TB therapy were excluded from the antibiotic counts. Data were analyzed using R ver. 3.5.1 [16]. Comparisons between groups were conducted using Chi-square tests or the Fisher’s exact test, with *P* < 0.05 considered statistically significant. The survey protocol was approved by the relevant institutional review board (IRB) committees (KNH-University of Nairobi #P295/06/2017; applicable to all study hospitals, Washington State University #16183, Centers for Disease Control and Prevention reliance approval on KNH-University of Nairobi IRB). Written informed consent was obtained from adult respondents and from guardians of children prior to their enrollment. No incentives were provided to study respondents.

## Results

### Hospital and patient characteristics

The survey lasted eight days at the Kenyatta National Hospital (KNH) in Nairobi, six days at Coast Provincial General Hospital (CPGH) in Mombasa and ten days at the Moi Teaching and Referral Hospital (MTRH) in Eldoret. KNH reported having 2,075 beds with 83,138 total admissions in 2016, CPGH had 700 beds with 24,205 total admissions (2016) and MTRH had 939 beds with 44,229 total admissions (2016). Of 1,071 patients included in the study, S3 File, 53% were female and 67% were aged ≥18 years. Twenty-eight percent of the surveyed patients had been hospitalized within the last 90 days and 20% had documented transfers from another healthcare facility, Table 1. The median duration of patient hospitalization until the survey day was 10 days at KNH, 4 days at CPGH and 9 days at MTRH.

**Table 1:** Participant characteristics at Kenyatta National Hospital (KNH), Coast Provincial General Hospital (CPGH) and Moi Teaching and Referral Hospital (MTRH), 2017–2018, Kenya.

	KNH	CPGH	MTRH	Total
	n (%)	n (%)	n (%)	n (%)
Patients surveyed	579	229	263	1,071
Females	298 (51)	129 (56)	136 (52)	563 (53)
Males	281 (49)	100 (44)	127 (48)	508 (47)
Age distribution				
Neonates (≤ 28 d)	46 (8)	31 (14)	19 (7)	96 (9)
Infants (≥ 1 –≤ 11 m)	34 (6)	27 (12)	17 (7)	78 (7)
Children (1–4 y)	43 (7)	13 (6)	14 (5)	70 (7)
Children (5–17 y)	59 (10)	13 (6)	33 (13)	105 (10)
Adults (≥18 y)^a^	397 (69)	145 (63)	180 (68)	722 (67)
Previously hospitalized^b^	149 (26)	44 (19)	105 (40)	298 (28)
Patients transferred in^c^	126 (22)	27 (12)	57 (22)	210 (20)
Patients by ward				
Critical care (ICU, HDU)	10 (2)	4 (2)	3 (1)	17 (2)
Medical	113 (20)	54 (24)	44 (17)	211 (20)
Obstetrics/gynecology	91 (16)	54 (24)	32 (12)	177 (17)
Pediatric	123 (21)	66 (29)	76 (29)	265 (25)
Private^d^	34 (6)	-	27 (10)	61 (6)
Specialized care^e^	24 (4)	-	29 (11)	53 (5)
Surgical	183 (32)	51 (22)	52 (20)	286 (27)

^a^Includes five adult patients whose ages could not be determined from hospital records/staff

^b^Patients with documented hospitalizations within 90 days prior to current hospitalization

^c^Patients with documented transfers from other healthcare facilities

^d^Private ward patients have personal physicians; CPGH did not have this ward type or a specialized care ward

^e^At KNH: Burns, oncology, and renal wards; MTRH: Cardiac care, eye, mental health, and neurology wards.

ICU: Intensive care unit; HDU: High-dependency Unit.

### Antibiotic use

Forty-six percent (489/1071) of all patients received at least one antibiotic at the time of the survey, Table 2. The proportion of patients receiving antibiotics did not differ significantly among the surveyed hospitals (*P* = 0.26). Antimicrobial use was higher among children <5 years compared to other age groups (70% (150/224) vs 40% (339/847); *P* < 0.001 Table 2. Critical care (82%, 14/17 patients) and pediatric wards (59%, 155/265) had the highest proportion of patients receiving antibiotics, Table 3. Among patients on antibiotics, 53% (258/489) were using one antibiotic, while 40% (197/489), 7% (32/489) and 0.4% (2/489) were using two, three and four antibiotics, respectively.

**Table 2:** Distribution of antibiotic use by patient sex and age at Kenyatta National Hospital (KNH), Coast Provincial General Hospital (CPGH) and Moi Teaching and Referral Hospital (MTRH), 2017–2018, Kenya.

	KNH	CPGH	MTRH	Total
	n/N (%)	n/N (%)	n/N (%)	n/N (%)
Patients on antibiotics	246/579 (43)	119/229 (52)	124/263 (47)	489/1071 (46)
Females	141/298 (47)	66/129 (51)	66/136 (49)	273/563 (49)
Males	105/282 (37)	53/100 (53)	58/127 (46)	216/508 (43)
Distribution by age				
Neonates (≤ 28 d)	35/46 (76)	22/31 (71)	8/19 (42)	65/96 (68)
1 m to 4 y	36/77 (47)	30/40 (75)	19/31 (61)	85/148 (57)
5–10 y	12/35 (34)	1/7 (14)	9/17 (53)	22/59 (37)
11–20 y	21/50 (42)	8/18 (44)	7/25 (28)	36/93 (39)
21–30 y	55/116 (47)	19/41 (46)	21/45 (47)	95/202 (47)
31–40 y	51/122 (42)	23/41 (56)	16/43 (37)	90/206 (44)
41–50 y	11/56 (20)	10/23 (44)	15/33 (46)	36/112 (32)
51–60 y	9/30 (30)	3/14 (21)	15/26 (58)	27/70 (39)
>60 y	14/45 (31)	3/12 (25)	14/23 (61)	31/80 (39)
Not specified^a^	2/2 (100)	0/2(0)	0/1 (0)	2/5 (40)

^a^Adult patients whose ages could not be determined from hospital records or from hospital staff. The number of patients receiving antibiotics is denoted by ‘n’.

**Table 3:** Distribution of antibiotic users (n = 489) by ward type at Kenyatta National Hospital (KNH), Coast Provincial General Hospital (CPGH) and Moi Teaching and Referral Hospital (MTRH), 2017–2018, Kenya.

	KNH	CPGH	MTRH	Total
	n/N (%)	n/N (%)	n/N (%)	n/N (%)
Critical care (ICU, HDU)	8/10 (80)	4/4 (100)	2/3 (67)	14/17 (82)
Medical	44/113 (39)	18/54 (33)	18/44 (41)	80/211 (38)
Obstetrics/gynecology	50/91 (55)	23/54 (43)	11/32 (34)	84/177 (48)
Pediatric	69/123 (56)	49/66 (74)	37/76 (49)	155/265 (59)
Private^†^	14/36 (39)	-	20/27 (74)	34/63 (54)
Specialized care	4/23 (17)	-	4/29 (14)	8/52 (15)
Surgical	57/183 (31)	25/51 (49)	32/52 (62)	114/286 (40)

^†^Private ward patients have personal physicians. The number of patients receiving antibiotics is denoted by ‘n’.

ICU: Intensive care unit; HDU: High dependency unit.

**Fig 1:**
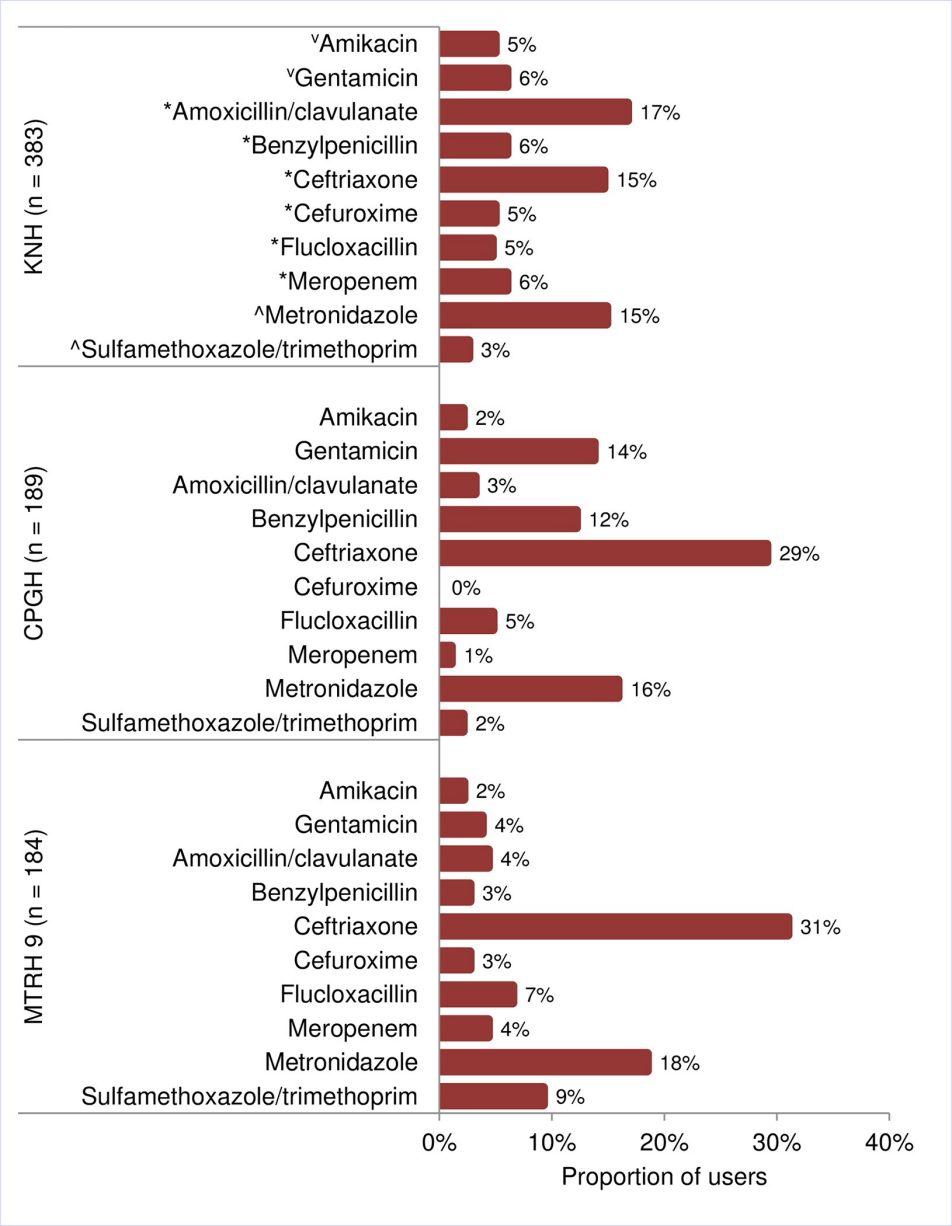
Commonly administered antibiotics to patients at Kenyatta National Hospital (KNH), Coast Provincial General Hospital (CPGH) and Moi Teaching and Referral Hospital (MTRH), 2017–2018, Kenya. Proportions are based on the total count of antibiotics in use by patients who were receiving antibiotics during the survey (n). Antibiotics ordered by class; ⱽAminoglycosides, *β-lactam antibiotics and ^Other classes.

Seven-hundred and fifty-six (756) separate antibiotic prescriptions were recorded (KNH = 383, CPGH = 189 and MTRH = 184). Forty-four percent (167/383) of antibiotics prescribed at KNH had at least one missed dose recorded, compared with 52% (98/189; *P* > 0.05) at CPGH and 33% (61/184; *P* = 0.02) at MTRH. There was some variability in the antibiotics used by the surveyed hospitals. Amoxicillin/clavulanate was the most used antibiotic at KNH (17%, 64/383 prescriptions) while ceftriaxone, a third-generation cephalosporin, was the most used at CPGH (29%, 55/189) and MTRH (31%, 57/184), Fig 1. Metronidazole was the second most used antibiotic in all the three hospitals, where it represented 15% (57/383) of use at KNH, 16% (30/189) at CPGH and 18% (34/184) at MTRH. Meropenem was the only reported carbapenem, representing <7% of prescriptions for any single hospital (Fig 1).

Almost one-third (34%, 258/756) of antibiotic prescriptions were in pediatric wards, where benzylpenicillin (19%, 50/258), gentamycin (19%, 50/258) and ceftriaxone (17%, 43/258) were the most prescribed antibiotics, S1 Table. Antibiotics used in surgical wards accounted for 22% (166/756) of total prescriptions, S1 Table. with ceftriaxone (27%, 45/166) being the most prescribed antibiotic across all hospital surgical wards. Obstetrics/gynecology (19%, 147/756) ward had the third highest antibiotic prescriptions with metronidazole (35%, 52/147) being the main antibiotic, S1 Table.

More than half of all patients with a documented catheter insertion (51%, 446/871), tube insertion (55%, 180/325), or surgical procedure (54%, 182/335) during the current admission were on an antibiotic. Comparatively 22% (43/200; *P* < 0.01), 41% (309/746; *P* < 0.01), and 42% (307/736; *P* < 0.01) of those who did not have a catheter insertion, tube insertion or surgical procedure, respectively, were using an antibiotic. Among surgical patients receiving antibiotics, 71% (130/182) had undergone an invasive surgery during the current admission, while 22% (40/182) and 7% (12/182) had minimally invasive- and non-invasive surgeries, respectively. Of the 484 patients whose HIV status were documented, 61% (50/82) of those with a HIV-positive status were receiving an antibiotic compared with 45% (182/402; *P* = 0.01) of HIV-negative patients.

### Prescription indications

Of the 489 patients using antibiotics, 90% (442/489) had a single documented diagnosis, 8% (39/489) had two diagnoses and 2% (8/489) had three diagnoses. Twenty-two percent (95/442) of patients with a single diagnosis were given antibiotics for prophylaxis and 32% (143/442) for an unspecified infectious condition (i.e., no information provided). The remaining 46% (204/442)—who constituted 86, 68 and 50 patients at KNH, CPGH and MTRH, respectively—were taking antibiotics for the treatment of a specified/documented infectious diagnosis. The five most common infections were soft-tissue infections (18%, 37/204), clinical sepsis (17%, 35/204), pneumonia (15%, 31/204 cases), central nervous system infections (13%, 27/204) and obstetric or gynecological infections (10%, 20/204).

### Bacterial culture and antibiotic susceptibility testing

Twenty-seven percent (27%; 56/204) of patients receiving an antibiotic and with a single specified infectious diagnosis had culture tests ordered. There were more tests ordered at KNH (34%; 29/86) than at CPGH (26%; 18/68) or MTRH (18%; 9/50) but the differences were not statistically significant (*P* > 0.05). Thirty-four samples were collected for culture from the 29 patients at KNH, 21 from the 18 patients at CPGH and 10 from the 9 patients at MTRH, for a cumulative 65 samples. Cerebrospinal fluid (25%, 16/65) and pus swabs (25%, 16/65) were collected most frequently across all hospitals. Other common samples included blood (15%, 10/65), urine (11%, 7/65), stool (9%, 6/65) and sputum (8%, 5/65).

The presumptive diagnoses for which culture tests were most ordered included CNS infections (23%; 15/65), pneumonia (23%; 15/65), soft-tissue infections (22%, 14/65), and clinical sepsis (9%, 6/65). For these conditions, culture results were available for 67% (10/15), 73% (11/15), 79% (11/14), and 50% (3/6) of samples submitted for the above diagnoses, respectively. Antibiotic susceptibility testing data were not collected during the survey at KNH and were scant at CPGH and MTRH, and were, therefore, excluded from the analysis.

All hospitals had a clinical microbiology laboratory staffed with Kenya Medical Laboratory Technicians and Technologists Board-certified microbiologists. KNH microbiology laboratory conducted 3,930 specimen cultures and 1,509 antibiotic susceptibility tests (ASTs), in the 3-month period preceding the survey compared with 518 cultures and 185 ASTs at CPGH, and 1,200 culture tests and 650 ASTs at MTRH. Only CPGH reported experiencing periodic stockouts of AST reagents.

### Antimicrobial stewardship and infection control programs

KNH and MTRH had Infection Prevention and Control (IPC) committees, and minutes available from meetings held within the six months preceding the survey. Only KNH had an antimicrobial stewardship committee. It was led by an infectious disease physician and provided formal guidelines to assist clinicians in making empirical decisions about antibiotic use. No hospital required clinicians to provide a rationale for prescribing antibiotics or to re-assess antibiotic prescriptions after 48 hours of the initial order. At KNH, 28% (106/383) of antibiotic prescriptions had no “stop” or “review” dates indicated, compared to 37% (70/189) at CPGH and 72% (133/184) at MTRH (*P* < 0.023). At the time of the study, no hospital required antimicrobial prescription pre-approval from another physician or pharmacist before administration or required that medical records include a rationale for antibiotic prescriptions. Further, no hospital conducted audits or reviews on the choice and duration of surgical antimicrobials used for prophylaxis. None of the hospitals’ laboratories produced an antimicrobial susceptibility report for the year preceding the survey.

## Discussion

The overall prevalence of the use of antibiotics in the three hospitals surveyed (46%) was lower than reports from other facilities in Kenya and Africa but higher than what is reported in high income countries/other regions. Recent point-prevalence survey results in two public referral hospitals in Kenya reported 68% [17] and 55% prevalence [18] of antibiotic use and the 2015 Global Point-prevalence Survey (GPPS) reported that 50% of hospitalized adults surveyed in Africa (5 countries, 12 hospitals) were using an antibiotic at the time of the survey [19]. Separate multi-facility surveys within Africa have reported antibiotic use prevalence estimates of 56% (9 hospitals in Nigeria) [20], 59% (18 hospitals in Egypt) [21] and 65% (39 hospitals in Benin) [22]. Comparatively, Europe had a prevalence of 32% (32 countries, 215 hospitals), the Americas had 38% (6 countries, 43 hospitals), and Asia had 39% (15 countries, 56 hospitals) [19].

The beta-lactam antibiotics amoxicillin/clavulanate and ceftriaxone were the most used antibiotics in this survey, as has been reported in other surveys in Kenya [12, 18] and in other African countries [19]. Meropenem use was limited (<7%) consistent with other surveys in Kenya [18], and was lower than that reported by other countries in Africa [19]. This may be because meropenem and other carbapenems are not included in Kenya’s Essential Medicines List [23] and are reserved for the treatment of recalcitrant infections under the guidance of an infectious disease specialist [24]. Antibiotic use was significantly higher among patients with catheter insertions, tube insertions or surgical procedures than those without, suggesting that these patients may have more severe diseases or that these procedures are sources of nosocomial infections.

We found substantive evidence that antibiotic use may not be optimal in the surveyed hospitals. Missed antibiotic doses, low use of specimen cultures to guide therapy, relatively high numbers of antibiotic prescriptions, particularly in the pediatric and surgical populations, and prescription of a broad-spectrum antibiotic like ceftriaxone without providing a rationale or reviewing these prescriptions were prevalent. Addressing all of these areas will be challenging, particularly given the limited resources for stewardship activities. Use of CDC [25] and WHO [26] guidance documents can direct initial AMS activities based on resource availability including routine antibiotic review, monitoring of antibiotic use, and use of antibiograms.

We also identified several areas for improvement that may be less resource intensive. The lack of locally generated antibiograms and use of clinical guidelines might be contributing to sub-optimal clinical care and worsening rates of AMR; development of locally appropriate treatment guidelines can help. While it is not possible from this study to determine the impact of oversight committees (e.g., drugs and therapeutics, infection prevention and control, and AMS), clarifying the committees’ roles and responsibilities in optimizing antibiotic use within these hospitals could contribute to improvements in AMS.

An aspect that stood out from the survey was the infrequent use of laboratory diagnostics to guide prescribing practices; few patients with suspected infectious syndromes had a culture ordered. Given that patients bear the cost of diagnostic testing and that fewer than 20% have health insurance coverage [26], clinicians may opt for empiric antibiotic therapy to help patients manage the cost of treatment, influencing their antibiotic prescription decisions. Solutions that prioritize laboratory testing to support antimicrobial prescribing practices, and those that eliminate the barriers faced by clinicians in using laboratory services are needed. For example, the cost of diagnostics could be subsidized to encourage their use. Application of regulatory frameworks that incentivize appropriate antibiotic use, require review of prescription practices, or provide oversight committees with more authority to influence practitioner behavior should be considered.

Our survey had several limitations. Firstly, we focused on large government tertiary facilities whose data may not be generalizable to smaller or private facilities within Kenya. Secondly, we did not collect data from some wards and cannot determine the exact percentage of the hospital population included in the study. We, therefore, caution against extrapolating these findings to wards that were excluded. Thirdly, for about half of the surveyed participants, we could not link specific antibiotics with a diagnosis or identify the rationale for their use either due to poor documentation or limited sample sizes for some comparisons.

This descriptive study contributes to the growing data on antibiotic use in tertiary care hospitals in Kenya and can be used by health facilities and the Ministry of Health to institute or improve AMS programs, policies, and guidelines. It also highlights important considerations that may be relevant in other low- or middle-income countries. Future research should focus on exploring the rationale underlying antibiotic use by specialties or specific classes of antibiotics, evaluating the appropriateness of antibiotic prescriptions, and identifying the determinants of effective AMS programs, drug and therapeutics committees and infection prevention and control strategies.

## Supporting information

S1 File Point-prevalence survey form used for hospital level data collection. (DOCX)

S2 File Point-prevalence survey form used for patient level data collection. (DOCX)

S3 File Point-prevalence survey data. (XLSX)

S4 File Inclusivity in global research. (DOCX)

S1 Table Antibiotic prescriptions in the three survey hospitals, distributed by the type of inpatient ward. (DOCX)
