# Supplementary material for: Point-prevalence survey of antibiotic use at three public referral hospitals in Kenya
Source: PLoS One. 2022 Jun 16;17(6):e0270048. doi: 10.1371/journal.pone.0270048 (PMC9202938; doi:10.1371/journal.pone.0270048)
Supplement: S1 File — (DOCX) [file pone.0270048.s001.docx]

**S1 file: Point-prevalence survey form used for hospital level data collection**

**Hospital data form**

Do not leave any field in this form blank; all details are required.

Date of data collection (dd / mmm / yy): From: _ _ / _ _ _ / _ _ To: _ _ / _ _ _ / _ _

Name of the data collector(s) (Print In capital letters) ___________________________________________

**A. General information**

Name of the Hospital (enter name) ___________________________________________

Current bed capacity (enter number) _____________ beds

Annual admission rate (enter number) _____________ in-patients/year

*Instructions to respondent*: Please respond “**YES**” or “**NO**” or provide a number (as appropriate) for each of the following questions.

**B. Infrastructure**

1) Does your facility have an administratively recognized antibiotic stewardship program or committee? Yes ___ No ___

a) Are stewardship committee minutes available from the past 6 months? Yes ___ No ___

b) Are any staff dedicated to supporting clinical decisions about antibiotic use? Yes ___ No ___

c) Is a physician identified as a leader for antimicrobial stewardship activities? Yes ___ No ___

d) Is a pharmacist involved with the antibiotic stewardship program or committee? Yes ___ No ___

e) Does your facility have information technology support for antibiotic stewardship activities? Yes ___ No ___

2) Does a board-certified microbiologist work at your facility? Yes ___ No ___

3) Does your facility have a clinical microbiology lab? Yes ___ No ___

a) How many culture tests were conducted in the past 3 months to identify clinical isolates? ________ tests

b) Have the necessary culture reagents been available during the last 3 months? Yes ___ No ___

i) Number of days in the past 3 months when cultures could not be performed due to lack of reagents? Yes ___ No ___

c) How many antibiotic susceptibility tests were conducted in the past 3 months? ________ tests

d) Have the necessary antibiotic susceptibility reagents been available during the last 3 months? Yes ___ No ___

e) Number of days in the last 3 months when susceptibility testing could not be done due to:

(1) Lack of reagents? _________ days

(2) Equipment failure? _________ days

4) Are any formal antibiotic use guidelines available in the ward? Yes ___ No ___

5) Is there an Infection Prevention & Control Committee in the hospital? Yes ___ No ___

a) Are minutes available from this committee from the past six months? Yes ___ No ___

6) Is there a Drugs and Therapeutics Committee in the hospital? Yes ___ No ___

a) Are minutes available from this committee from the past six months? Yes ___ No ___

7) Is there a current/updated Essential Drug List available/accessible in the ward? Yes ___ No ___

**C. Policy and Practice**

8) At the ward level, are there formal guidelines to assist with empirical decisions about antibiotic use? Yes ___ No ___

a) Are these guidelines based on local antimicrobial susceptibility test results? Yes ___ No ___

9) In the past 12 months has training been providing regarding local antibiotic resistance patterns and concerns? Yes ___ No ___

10) Does your facility have a written policy requiring prescribers to provide a rationale for prescribing antibiotics? Yes ___ No ___

11) Are separate reviews by another physician or pharmacist routinely required before prescribing antibiotics? Yes ___ No ___

12) Is there a formal review process to re-assess antibiotic prescriptions after 48 hours of an initial order? Yes ___ No ___

**D. Monitoring and Feedback**

13) Has your facility produced an antimicrobial susceptibility report in the past year? Yes ___ No ___

14) Does your facility produce an annual report about antibiotic stewardship? Yes ___ No ___

15) Does your facility confirm that medical records consistently include a rationale for antibiotic prescriptions? Yes ___ No ___

16) Does your facility audit or review surgical antimicrobial prophylaxis choice and duration? Yes ___ No ___

17) Are results of antimicrobial audits or reviews communicated directly with prescribers? Yes ___ No ___
