# Supplementary material for: Point-prevalence survey of antibiotic use at three public referral hospitals in Kenya
Source: PLoS One. 2022 Jun 16;17(6):e0270048. doi: 10.1371/journal.pone.0270048 (PMC9202938; doi:10.1371/journal.pone.0270048)
Supplement: S2 File — (DOCX) [file pone.0270048.s002.docx]

**S2 file: Point-prevalence survey form used for patient level data collection**

**Patient Data form**

**SECTION 1**

**Patient data**

Participant ID __________________________________

Research Assistant code (*Appendix I*) __________________________________

Date of data collection __________________________________

Name of the Hospital __________________________________

Ward group __________________________________

Ward Name _________________________________

Did the participant give informed consent? Yes___ No___

Inpatient file number (IP) __________________________________

Admission date __________________________________

Age __________________________________

(Adults and older children, state in Years (e.g. 34))

(Infants, state in Months (e.g. 9))

(Neonates, state in days (e.g. 16))

Sex M____ F____

Any hospitalization in the last 90 days, excl. current Yes___ No___ Not documented (ND)___

Was participant transferred from another hospital? Yes___ No___ ND____

Any catheterization on participant during this admission? Yes___ No___ ND____

If yes, number of catheterizations _______ Indicate number

If yes, type of catheterization _______________ Specify type

Any intubation on participant during current admission? Yes___ No___ ND____

If yes, number of intubations _______ Indicate number

If yes, type of intubation _______________ Specify type

Any surgery done during this admission? Yes___ No___

If yes, which type (most recent surgery)? Invasive_______ Minimally Invasive Procedure_____ Non-Invasive_____

*(See definitions and examples; Appendix I)*

Was a malaria test done? Yes___ No___

What was the result? Positive____ Negative____

HIV Status Positive____ Negative____ ND____

If positive, is the participant on HAART? Yes___ No___ ND

Is the participant on TB treatment? Yes___ No___

Is the participant currently on any antibiotic other than for TB? Yes___ No___

**SECTION 2**

*To be completed only for participant currently on Antibiotic therapy other than for* TB (Do not proceed if the participant is treated only for TB)

Number of indications for which antibiotics were given _______ (Indicate number)

Type of indication (*use codes provided in Appendix II)* _______ (Indicate code)

Number of antibiotics the participant is on _______ (Indicate number)

**Enter up to five antibiotics**

ATC code for Antibiotic: 1 (*use codes provided in Appendix III*)

Start date ____________________

Dose per administration ____________________

Unit of measure ____________________

Route of administration PO___ IV____ IM____ Other____

Frequency of administration STAT__ OD___ BID___ TID___ QID___Q4H___Other___

Is an antibiotic stop/review date documented? Yes___ No___

Is the antibiotic being used for Prophylaxis? Yes___ No___ ND___

If for Prophylaxis, specify type: Medical ____ Surgical_____

Prophylaxis duration? One single dose_________

Multiple doses within 24 hours______

More than 1 day______

For which indication is the antibiotic being given? Indication 1__ 2___ 3___ 4___ 5____ ND___

Is the drug prescribed in its INN (generic name)? Yes___ No___

Is the drug in the Essential Medicines List (KEML)? Yes___ No___

No. of missed doses since antibiotic started __________________________________

(Count from date of initiation to current date how many doses were missed, state it as simple count. If 6 doses missed capture as 6; if none state 0.)

**Culture Tests**

Was Culture ordered Yes___ No___

Culture tests done during the current admission _______ (Indicate number)

**Enter up to three culture tests**

Specimen used _____________

Are culture results available? Yes___ No___

Was antimicrobial susceptibility testing (AST) ordered? Yes___ No___

Are AST results available? Yes___ No___

**Appendix I: Definition of Surgery Types**

**Invasive surgery**

Traditional open surgery where incisions -larger than minimally invasive. Include

- - - - - Laparatomy
        - Thoracotomy
        - Caesarian Section
        - Open Heart Surgeries
        - Open othorpaedic surgeries
        - Hysterectomy and Tubal operations not done through endoscopy or laparascopy
        - Tooth extractions, gum surgery or dental implants

**Minimally invasive surgery**

Minimally invasive surgery is a surgical procedure in which operations are performed through very small incisions. These include:

- - - - - Endoscopic procedures
        - Hysteroscopy
        - Laparoscopy, thoracoscopy, arthroscopy
        - Dilatation and curettage
        - Minimally Invasive Coronary Artery Bypass
        - Angioplasty
        - Dermatological procedures e.g., biopsy, excision using local anesthesia

**Non-invasive surgery**

Non-invasive procedures including the following:

- - - - - Obstetric procedures: peri-delivery/labour
        - Dental extraction
        - Transurethral resection of prostate
        - Incision and drainage of abscess with secondary closure
        - Any diabetic forefoot amputation with healing by secondary intention
        - Any other operation where healing is by secondary intention
        - Tonsillectomy
        - Application of external fixator/Olizarov
        - Extraventricular drain
        - Hysteroscopic removal of fibroids: evacuation of retained products of conception

**Appendix II: ATC codes for antibiotics**

| Amikacin (J01GB06) | Daptomycin (J01XX09) | Piperacillin (J01CA12) |
| --- | --- | --- |
| Amoxicillin (J01CA04) | Demeclocycline (J01AA01) | Piperacillin and enzyme inhibitor (J01CR05) |
| Amoxicillin and enzyme inhibitor (J01CR02) | Doxycycline (J01AA02) | Polymyxin B (A07AA05) |
| Ampicillin (J01CA01) | Ertapenem (J01DH03) | Procaine benzylpenicillin (J01CE09) |
| Ampicillin and enzyme inhibitor (J01CR01) | Erythromycin (J01FA01) | Rifampicin (J04AB02) |
| Ampicillin, combinations (J01CA51) | Flucloxacillin (J01CF05) | Secnidazole (P01AB07) |
| Aztreonam (J01DF01) | Fosfomycin (J01XX01) | Sparfloxacin (J01MA09) |
| Azithromycin (J01FA10) | Fusidic acid (J01XC01) | Spectinomycin (J01XX04) |
| Benzathinebenzylpenicillin (J01CE08) | Gentamicin (J01GB03) | Streptomycin (parenteral) (J01GA01) |
| Benzathinephenoxymethylpenicillin (J01CE10) | Griseofulvin D01BA01) | Streptomycin, combinations (A07AA54) |
| Benzylpenicillin (J01CE01) | Imipenem and enzyme inhibitor (J01DH51) | Sulbactam (J01CG01) |
| Cefaclor (J01DC04) | Kanamycin (J01GB04) | Sulfadiazine (J01EC02) |
| Cefadroxil (J01DB05) | Levofloxacin (J01MA12) | Sulfadiazine and trimethoprim (J01EE02) |
| Cefalexin (J01DB01) | Linezolid (J01XX08) | Sulfamethoxazole (J01EC01) |
| Cefazolin (J01DB04) | Meropenem (J01DH02) | Sulfamethoxazole and trimethoprim (J01EE01) |
| Cefepime (J01DE01) | Metronidazole (oral, rectal) (P01AB01) | Sulfamethoxypyridazine (J01ED05) |
| Cefixime (J01DD08) | Metronidazole (parenteral) (J01XD01) | Sulfonamides, combinations with other antibacterials (excl. trimethoprim) (J01RA02) |
| Cefodizime (J01DD09) | Minocycline (J01AA08) |  |
| Cefoperazone (J01DD12) | Moxifloxacin (J01MA14) | Tazobactam (J01CG02) |
| Cefotaxime (J01DD01) | Nalidixic acid (J01MB02) | Teicoplanin (J01XA02) |
| Cefoxitin (J01DC01) | Neomycin (injection, infusion) (J01GB05) | Telavancin (J01XA03) |
| Cefpodoxime (J01DD13) | Nitrofurantoin (J01XE01) | Tetracycline (J01AA07) |
| Cefprozil (J01DC10) | Norfloxacin (J01MA06) | Ticarcillin (J01CA13) |
| Ceftazidime (J01DD02) | Nystatin (A07AA02) | Ticarcillin and enzyme inhibitor (J01CR03) |
| Ceftriaxone (J01DD04) | Ofloxacin (J01MA01) | Tigecycline (J01AA12) |
| Cefuroxime (J01DC02) | Ornidazole (oral) (P01AB03) | Tinidazole (oral, rectal) (P01AB02) |
| Chloramphenicol (J01BA01) | Ornidazole (parenteral) (J01XD03) | Tinidazole (parenteral) (J01XD02) |
| Ciprofloxacin (J01MA02) | Oxacillin (J01CF04) | Tobramycin (J01GB01) |
| Clarithromycin (J01FA09) | Paromomycin (A07AA06) | Trimethoprim (J01EA01) |
| Clindamycin (J01FF01) | Penicillins, combinations with other antibacterials (J01RA01) | Vancomycin (parenteral) (J01XA01) |
| Cloxacillin (J01CF02) |  |  |
| Colistin (injection, infusion) (J01XB01) | Phenoxymethylpenicillin (J01CE02) |  |

**Appendix III: Indication Codes for antibiotics given**

- CNS - Infections of the central nervous system (e.g., meningitis, brain abscess)
- EYE - Eye infections, e.g., endophthalmitis
- ENT - Infections of ear, nose, throat, larynx and mouth (upper respiratory tract excluding bronchus)
- BRON - Acute bronchitis or exacerbations of chronic bronchitis
- PNEU - Pneumonia (other than TB)
- CVS - Cardiovascular infections: (e.g., endocarditis, vascular graft.)
- GI - Gastrointestinal infections (e.g., salmonellosis, antibiotic-associated diarrhoea)
- IA - Intra-abdominal sepsis (between diaphragm and pelvic floor) including hepatobiliary and peritoneal cavity infections)
- SST - Soft tissue infections (e.g., cellulitis, wound, and deep soft tissue) not involving bone.
- BJ - Bone and joint infections (e.g., septic arthritis, prosthetic joint infections, osteomyelitis, etc.)
- CYS - Symptomatic lower urinary tract infection (urethra and bladder) e.g., cystitis
- PYE - Symptomatic upper urinary tract infection (ureter and kidney) e.g., pyelonephritis
- ASB - Asymptomatic bacteriuria (Presence of bacteria in urine without symptoms)
- OBGY - Obstetric or gynaecological infections (e.g., STDs in women, abortion related sepsis, post-partum sepsis, etc.)
- GUM - Prostatitis, epididymo-orchitis, and STD in men
- BAC - Laboratory-confirmed bacteremia (Positive blood culture with isolated bacteria)
- CSEP - Clinical sepsis (suspected bloodstream infection without lab confirmation/results are not available, no blood cultures collected or negative blood culture), excluding febrile neutropenia.
- FN - Febrile neutropenia or other form of manifestation of infection in immunocompromised host, e.g. HIV, chemotherapy, etc., with no clear anatomical site
- SIRS - Systemic Inflammatory Response Syndrome with no clear anatomical site of infection.
- UND - Completely undefined; site with no systemic inflammation
