# Supplementary material for: Point-prevalence survey of antibiotic use at three public referral hospitals in Kenya
Source: PLoS One. 2022 Jun 16;17(6):e0270048. doi: 10.1371/journal.pone.0270048 (PMC9202938; doi:10.1371/journal.pone.0270048)
Supplement: S1 Table — (DOCX) [file pone.0270048.s005.docx]

**S1 Table. Antibiotic prescriptions in the three survey hospitals, distributed by the type of inpatient ward**

|  | **Kenyatta National Hospital** | | | | | | | | **Coast Provincial General Hospital** | | | | | | | | **Moi Teaching and Referral Hospital** | | | | | | | | **All Hospitals** | | | | | | | |
| --- | --- | --- | --- | --- | --- | --- | --- | --- | --- | --- | --- | --- | --- | --- | --- | --- | --- | --- | --- | --- | --- | --- | --- | --- | --- | --- | --- | --- | --- | --- | --- | --- |
| **Antibiotic** | **1** | **2** | **3** | **4** | **5** | **6** | **7** | **N** | **1** | **2** | **3** | **4** | **5** | **6** | **7** | **N** | **1** | **2** | **3** | **4** | **5** | **6** | **7** | **N** | **1** | **2** | **3** | **4** | **5** | **6** | **7** | **N** |
| Amikacin | 0 | 15 | 2 | 0 | 0 | 0 | 2 | **19** | 0 | 3 | 1 | 0 | 0 | 0 | 0 | **4** | 0 | 3 | 0 | 0 | 0 | 1 | 0 | **4** | 0 | 21 | 3 | 0 | 0 | 1 | 2 | **27** |
| Amoxicillin | 1 | 1 | 0 | 0 | 0 | 0 | 1 | **3** | 3 | 0 | 1 | 0 | 0 | 0 | 0 | **4** | 0 | 0 | 1 | 0 | 1 | 0 | 0 | **2** | 4 | 1 | 2 | 0 | 1 | 0 | 1 | **9** |
| Amoxicillin/clavulanic acid | 34 | 6 | 11 | 6 | 2 | 6 | 0 | **65** | 2 | 0 | 3 | 1 | 0 | 0 | 0 | **6** | 0 | 0 | 2 | 1 | 1 | 4 | 0 | **8** | 36 | 6 | 16 | 8 | 3 | 10 | 0 | **79** |
| Azithromycin | 0 | 2 | 0 | 0 | 0 | 1 | 0 | **3** | 0 | 0 | 0 | 0 | 0 | 0 | 0 | **0** | 0 | 2 | 0 | 4 | 0 | 0 | 0 | **6** | 0 | 4 | 0 | 4 | 0 | 1 | 0 | **9** |
| Benzathinebenzylpenicillin | 0 | 1 | 0 | 0 | 0 | 0 | 0 | **1** | 0 | 0 | 0 | 0 | 0 | 0 | 0 | **0** | 0 | 0 | 0 | 0 | 0 | 0 | 0 | **0** | 0 | 1 | 0 | 0 | 0 | 0 | 0 | **1** |
| Benzylpenicillin | 0 | 22 | 0 | 0 | 0 | 1 | 0 | **23** | 0 | 23 | 0 | 0 | 0 | 0 | 0 | **23** | 0 | 5 | 0 | 0 | 0 | 0 | 0 | **5** | 0 | 50 | 0 | 0 | 0 | 1 | 0 | **51** |
| Cefazolin | 0 | 0 | 0 | 0 | 0 | 0 | 0 | **0** | 0 | 0 | 0 | 0 | 0 | 0 | 0 | **0** | 0 | 0 | 1 | 0 | 0 | 0 | 0 | **1** | 0 | 0 | 1 | 0 | 0 | 0 | 0 | **1** |
| Cefepime* | 0 | 0 | 0 | 0 | 0 | 0 | 0 | **0** | 0 | 5 | 0 | 0 | 0 | 0 | 0 | **5** | 0 | 4 | 0 | 0 | 0 | 0 | 0 | **4** | 0 | 9 | 0 | 0 | 0 | 0 | 0 | **9** |
| Cefixime* | 0 | 0 | 0 | 0 | 0 | 0 | 0 | **0** | 1 | 0 | 0 | 0 | 0 | 0 | 0 | **1** | 0 | 0 | 0 | 0 | 0 | 0 | 0 | **0** | 1 | 0 | 0 | 0 | 0 | 0 | 0 | **1** |
| Cefodizime | 0 | 2 | 0 | 0 | 0 | 0 | 0 | **2** | 0 | 0 | 0 | 0 | 0 | 0 | 0 | **0** | 0 | 0 | 0 | 0 | 0 | 0 | 0 | **0** | 0 | 2 | 0 | 0 | 0 | 0 | 0 | **2** |
| Ceftazidime | 0 | 6 | 0 | 2 | 0 | 0 | 0 | **8** | 0 | 3 | 0 | 0 | 0 | 0 | 0 | **3** | 0 | 0 | 0 | 0 | 0 | 0 | 0 | **0** | 0 | 9 | 0 | 2 | 0 | 0 | 0 | **11** |
| Ceftriaxone | 9 | 10 | 20 | 13 | 0 | 3 | 1 | **56** | 15 | 16 | 12 | 9 | 0 | 0 | 3 | **55** | 9 | 17 | 13 | 9 | 0 | 8 | 1 | **57** | 33 | 43 | 45 | 31 | 0 | 11 | 5 | **168** |
| Cefuroxime* | 7 | 0 | 7 | 1 | 0 | 4 | 0 | **19** | 0 | 0 | 0 | 0 | 0 | 0 | 0 | **0** | 0 | 0 | 4 | 0 | 0 | 1 | 0 | **5** | 7 | 0 | 11 | 1 | 0 | 5 | 0 | **24** |
| Chloramphenicol* | 0 | 0 | 0 | 0 | 0 | 0 | 0 | **0** | 0 | 0 | 0 | 1 | 0 | 0 | 0 | **1** | 0 | 0 | 0 | 0 | 0 | 0 | 0 | **0** | 0 | 0 | 0 | 1 | 0 | 0 | 0 | **1** |
| Ciprofloxacin | 0 | 0 | 2 | 3 | 1 | 0 | 2 | **8** | 0 | 1 | 1 | 2 | 0 | 0 | 0 | **4** | 0 | 0 | 0 | 0 | 0 | 1 | 0 | **1** | 0 | 1 | 3 | 5 | 1 | 1 | 2 | **13** |
| Clarithromycin | 0 | 0 | 0 | 7 | 0 | 0 | 0 | **7** | 0 | 0 | 0 | 0 | 0 | 0 | 0 | **0** | 0 | 0 | 1 | 0 | 0 | 0 | 0 | **1** | 0 | 0 | 1 | 7 | 0 | 0 | 0 | **8** |
| Clindamycin | 0 | 1 | 4 | 1 | 0 | 1 | 1 | **8** | 0 | 0 | 2 | 1 | 0 | 0 | 0 | **3** | 0 | 0 | 2 | 0 | 0 | 1 | 0 | **3** | 0 | 1 | 8 | 2 | 0 | 2 | 1 | **14** |
| Doxycycline | 0 | 0 | 0 | 0 | 0 | 0 | 0 | **0** | 0 | 0 | 0 | 0 | 0 | 0 | 0 | **0** | 0 | 0 | 0 | 1 | 1 | 0 | 0 | **2** | 0 | 0 | 0 | 1 | 1 | 0 | 0 | **2** |
| Erythromycin* | 1 | 5 | 0 | 0 | 0 | 0 | 0 | **6** | 1 | 0 | 0 | 0 | 0 | 0 | 0 | **1** | 0 | 0 | 0 | 0 | 0 | 0 | 0 | **0** | 2 | 5 | 0 | 0 | 0 | 0 | 0 | **7** |
| Flucloxacillin | 1 | 2 | 10 | 5 | 0 | 0 | 0 | **18** | 0 | 3 | 6 | 0 | 0 | 0 | 0 | **9** | 0 | 1 | 9 | 1 | 0 | 1 | 0 | **12** | 1 | 6 | 25 | 6 | 0 | 1 | 0 | **39** |
| Gentamicin | 0 | 22 | 0 | 0 | 0 | 1 | 0 | **23** | 0 | 24 | 2 | 0 | 0 | 0 | 0 | **26** | 0 | 4 | 2 | 0 | 0 | 1 | 0 | **7** | 0 | 50 | 4 | 0 | 0 | 2 | 0 | **56** |
| Levofloxacin | 0 | 0 | 5 | 1 | 1 | 0 | 1 | **8** | 0 | 0 | 0 | 1 | 0 | 0 | 0 | **1** | 0 | 0 | 0 | 1 | 0 | 1 | 0 | **2** | 0 | 0 | 5 | 3 | 1 | 1 | 1 | **11** |
| Meropenem* | 5 | 12 | 2 | 2 | 0 | 0 | 2 | **23** | 0 | 0 | 1 | 0 | 0 | 0 | 1 | **2** | 0 | 4 | 2 | 0 | 0 | 1 | 1 | **8** | 5 | 16 | 5 | 2 | 0 | 1 | 4 | **33** |
| Metronidazole | 23 | 8 | 18 | 4 | 0 | 1 | 3 | **57** | 21 | 2 | 3 | 3 | 0 | 0 | 1 | **30** | 8 | 6 | 11 | 4 | 1 | 4 | 0 | **34** | 52 | 16 | 32 | 11 | 1 | 5 | 4 | **121** |
| Nitrofurantoin | 0 | 0 | 0 | 1 | 0 | 0 | 0 | **1** | 1 | 0 | 0 | 0 | 0 | 0 | 0 | **1** | 0 | 0 | 0 | 0 | 0 | 0 | 0 | **0** | 1 | 0 | 0 | 1 | 0 | 0 | 0 | **2** |
| Penicillin + other antibiotic | 2 | 0 | 0 | 0 | 0 | 0 | 0 | **2** | 0 | 0 | 0 | 0 | 0 | 0 | 0 | **0** | 0 | 0 | 0 | 0 | 0 | 0 | 0 | **0** | 2 | 0 | 0 | 0 | 0 | 0 | 0 | **2** |
| Phenoxymethylpenicillin | 0 | 1 | 0 | 0 | 0 | 0 | 0 | **1** | 0 | 1 | 0 | 0 | 0 | 0 | 0 | **1** | 0 | 0 | 0 | 0 | 0 | 0 | 0 | **0** | 0 | 2 | 0 | 0 | 0 | 0 | 0 | **2** |
| Piperacillin + enzyme inhibitor* | 1 | 1 | 1 | 0 | 0 | 0 | 1 | **4** | 0 | 0 | 0 | 1 | 0 | 0 | 0 | **1** | 0 | 0 | 0 | 0 | 0 | 0 | 0 | **0** | 1 | 1 | 1 | 1 | 0 | 0 | 1 | **5** |
| Sulfamethoxazole/trimethoprim | 1 | 1 | 0 | 8 | 0 | 0 | 0 | **10** | 0 | 0 | 0 | 4 | 0 | 0 | 0 | **4** | 1 | 6 | 0 | 7 | 1 | 2 | 0 | **17** | 2 | 7 | 0 | 19 | 1 | 2 | 0 | **31** |
| Tazobactam* | 0 | 0 | 0 | 1 | 0 | 0 | 0 | **1** | 0 | 0 | 0 | 0 | 0 | 0 | 0 | **0** | 0 | 0 | 0 | 0 | 0 | 0 | 0 | **0** | 0 | 0 | 0 | 1 | 0 | 0 | 0 | **1** |
| Trimethoprim | 0 | 0 | 0 | 2 | 0 | 0 | 0 | **2** | 0 | 0 | 0 | 0 | 0 | 0 | 0 | **0** | 0 | 0 | 0 | 0 | 0 | 0 | 0 | **0** | 0 | 0 | 0 | 2 | 0 | 0 | 0 | **2** |
| Vancomycin | 0 | 3 | 0 | 0 | 0 | 0 | 2 | **5** | 0 | 2 | 2 | 0 | 0 | 0 | 0 | **4** | 0 | 2 | 2 | 0 | 0 | 0 | 1 | **5** | 0 | 7 | 4 | 0 | 0 | 0 | 3 | **14** |
|  | **85** | **121** | **82** | **57** | **4** | **18** | **16** | **383** | **44** | **83** | **34** | **23** | **0** | **0** | **5** | **189** | **18** | **54** | **50** | **28** | **5** | **26** | **3** | **184** | **147** | **258** | **166** | **108** | **9** | **44** | **24** | **756** |

Ward codes: 1. Obs/gyn; 2, Peds; 3, Surgical; 4, Medical; 5, Special units; 6, Private; 7, Critical care. *Not included in the 2016 Kenya Essential Medicines List.
